# Reducing the levels of indoxyl sulfate in patients undergoing dialysis: a promising approach to managing inflammation and the redox state of human serum albumin

**DOI:** 10.25122/jml-2023-0538

**Published:** 2024-08

**Authors:** Wesam Ahmed Nasif, Mohammed Hassan Mokhtar, Ashraf Abdelazeem Ewis, Hiba Saeed Al-Amodi, Abeer Shaker El Moursy Ali

**Affiliations:** 1 Department of Biochemistry, College of Medicine, Umm Al-Qura University, Makkah, Saudi Arabia; 2 Molecular Biology Department, Genetic Engineering and Biotechnology Research Institute, Sadat City University, Sadat City, Egypt; 3 Department of Public Health and Occupational Medicine, Faculty of Medicine, Minia University, El-Minia, Egypt; 4 Department of Pathology, College of Medicine, Umm Al-Qura University, Makkah, Saudi Arabia

**Keywords:** indoxyl sulfate, oxidized albumin, inflammatory markers, chronic kidney disease (CKD)

## Abstract

Indoxyl sulfate (IS) is one of the most potent uraemic toxins involved in the progression of chronic kidney disease (CKD) through the induction of inflammation and oxidative stress. This study assessed the potential benefits of reducing IS concentrations through dialysis treatment to improve renal function, inflammation, and oxidative stress. A prospective, observational cohort study of 50 patients with CKD undergoing dialysis treatment was conducted. IS levels, inflammatory markers (IL-6 and hs-CRP), and oxidative status (Cu/Zn-SOD) were measured using immunoenzymatic methods, and the albumin ratio (HNA/HMA) was assessed using high-performance liquid chromatography. Blood samples were collected at baseline and, at 8 weeks and 16 weeks after treatment. At baseline, patients with CKD had elevated levels of IS, renal function indicators, inflammatory markers (IL-6 and CRP), and oxidative markers (Cu/Zn-SOD and albumin ratio HNA/HMA). Dialysis treatment reduced IS levels, and a correlation among IS, renal function, and SOD levels (*P* < 0.0001) at 8 and 16 weeks was observed. The reduction in IS levels was associated with improved inflammatory marker levels (CRP and IL-6; *P* < 0.0001) and a significant decrease in the HNA/HMA ratio (*P* <0.0001) at 8 and 16 weeks. These associations strengthened over time. The results of this study suggest that IS levels may be a therapeutic target for improving outcomes in patients with CKD by improving renal function, inflammation, and oxidative stress. More research is needed to understand how IS contributes to CKD complications.

## INTRODUCTION

**Figure 1:**
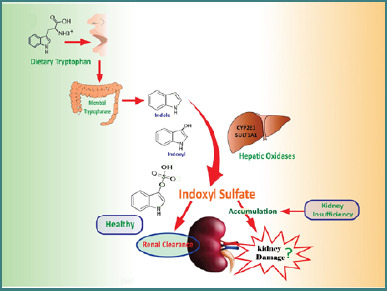
Pathway of IS production, accumulation, and its impact on kidney function in chronic kidney disease. Indoxyl sulfate (IS) is a uremic toxin that is produced in the gut by bacteria using tryptophan from food. IS is then absorbed into the bloodstream and transported to the liver, where it is sulphated by sulphotransferase (SULT)-1A1. In people with healthy kidneys, IS is excreted in the urine. However, in people with chronic kidney disease (CKD), IS excretion is reduced, leading to its accumulation in the blood. This buildup of IS can damage the kidneys even further, although whether this is causative is presently unclear. The illustrations adapted from the National Institute of Diabetes and Digestive and Kidney Diseases (NIDDK), National Institutes of Health (NIH), and are reproduced under a creative commons license.

Indoxyl sulfate (IS) is a uraemic toxin that accumulates in the blood of people with kidney disease. It is produced by gut bacteria and converted to indoxyl by the liver. Indoxyl is then conjugated with sulfate by the enzyme sulfotransferase to form IS [1]. It is normally cleared by the kidneys, but in patients with kidney disease, it can accumulate in the blood, as shown in Figure 1 [2]. High levels of IS have been linked to cardiovascular disease, mortality, and accelerated progression of chronic kidney disease (CKD) [3].

Indoxyl sulfate has been implicated in several cellular processes that may contribute to CKD and cardiovascular diseases (CVD), including oxidative stress, inflammation, endothelial dysfunction, and fibrosis. Studies have suggested that IS can induce oxidative stress by generating reactive oxygen species (ROS) and reducing antioxidant activity [4,5]. This oxidative stress can damage cellular components, such as DNA, proteins, and lipids. In addition, IS also activates several proinflammatory pathways in the kidneys, including the nuclear factor-kappa B (NF-κB) and toll-like receptor 4 (TLR4) pathways. These pathways stimulate the release of proinflammatory cytokines and chemokines, such as interleukin-6 (IL-6) and tumor necrosis factor-alpha (TNF-α), which promote inflammation and endothelial dysfunction, recruit immune cells to the site of injury and exacerbate the inflammatory response. Chronic inflammation contributes to renal fibrosis and scarring, which impairs renal function and accelerates the progression of CKD [6,7].

Despite the growing body of evidence linking IS to various pathological processes, the mechanisms by which IS exerts its toxic effects are not fully understood [6]. However, several strategies to reduce IS levels have been proposed in patients with CKD, including dietary interventions, probiotics, and pharmacological agents. Some studies suggested that reducing the dietary intake of tryptophan and increasing fiber consumption may reduce IS production by gut bacteria. Probiotics have also been proposed as a potential therapy for reducing IS levels, as they can modulate gut microbiota composition and reduce IS production. In addition, several pharmacological agents, such as AST-120 and sevelamer, have been shown to reduce IS levels in patients with CKD [7,8].

Studies have shown that hemodialysis can effectively remove IS from the blood, decreasing its levels [9]. However, the effectiveness of hemodialysis in removing IS may depend on several factors, including the duration and frequency of dialysis sessions, the type of dialyzer used, and the patient's residual kidney function [9,10]. Therefore, reducing the levels of IS through hemodialysis may have potential benefits in reducing the risk of complications in patients with CKD and end-stage renal disease (ESRD). To thoroughly comprehend the role of IS in these patients and the efficacy of hemodialysis in reducing its levels and enhancing outcomes, additional research is needed [11]. The current study aimed to assess and compare the concentrations of indoxyl sulfate in individuals with chronic kidney disease undergoing hemodialysis treatment. Additionally, this study aimed to explore the relationships between IS and markers of renal function, inflammation, and oxidative stress.

## MATERIAL AND METHODS

Blood samples were collected from 50 patients (36 men and 14 women) with CKD undergoing dialysis. The ages of the participants ranged from 26 to 62 years. Samples were obtained before and after dialysis sessions, referred to as the prior-hemodialysis (prior-HD) group and the post-hemodialysis (post-HD) group, respectively, resulting in 150 sample observations. CKD was diagnosed clinically based on the Kidney Disease: Improving Global Outcomes (KDIGO) classification guidelines [12]. Patients with active infections, pregnancy, chronic liver diseases, autoimmune diseases, or those unable to provide written consent were excluded from the study. All included patients were clinically stable and agreed to cooperate with hospital personnel. Prior to dialysis treatment, an initial blood sample was obtained from each patient. Two additional blood samples were collected at follow-up visits after 8 and 16 weeks. The serum samples were immediately stored at -80 °C until analysis.

### Assessment of biochemical markers

Creatinine levels were assessed using the Jaffe reaction, a colorimetric method that measures the amount of creatinine in a sample by its ability to form a yellow-orange complex with picric acid in an alkaline solution. The color intensity is proportional to the amount of creatinine in the sample. Urea levels were detected using the diacetyl monoxime colorimetric method and the Berthelot reaction, where urease converts urea to ammonia, which is then coupled with 2-oxoglutarate and nicotinamide adenine dinucleotide (NADH) in the presence of glutamate dehydrogenase (GDH) to form L-glutamate and NAD. The decrease in NADH absorbance is proportional to the urea concentration.

### Measurement of circulating indoxyl sulfate

Circulating indoxyl sulfate, a uremic toxin, was quantified using a quantitative sandwich ELISA kit (human indoxyl sulfate ELISA kit, Cat. no. MBS019983) [13]. In this assay, 50 µL of sample diluent and 100 µL of HRP-conjugated reagent were added to 100 µL of serum sample in a designated well. The plate was then incubated at 37 °C for 60 minutes. After incubation, a chromogen solution was added, and the plate was incubated in the dark for 15 minutes. The reaction was stopped, and the color changed from blue to yellow. The optical density (OD) of each well was measured at 450 nm using a microplate reader within 15 minutes of adding the stop solution. The detection range of this kit is 3.12-100 µg/mL, and the sensitivity is 1.0 µg/mL.

### Measurement of inflammatory biomarkers

C-reactive protein (CRP) levels in patients with hemodialysis (HD) were measured using the hs-CRP ELISA method (Diagnostic Automation/Cortez Diagnostics, CA 91302). This method utilizes a solid phase based on the principle of ELISA [13]. A conjugated HRP goat anti-CRP antibody was used for detection, and the concentration of CRP was determined spectrophotometrically at 450 nm. The intensity of the color is proportional to the concentration of CRP.

### Assessment of interleukin-6 levels

Human interleukin-6 (IL-6) levels were quantitatively measured using a standard sandwich ELISA kit. Serum samples and standards were added to precoated 96-well plates, followed by biotinylated polyclonal anti-IL-6 antibody and avidin-biotin-peroxidase complex. After washing to remove unbound conjugates, the HRP substrate TMB was added, resulting in a blue product that turned yellow upon adding an acidic stop solution. The absorbance of the yellow color at 450 nm was directly proportional to the human IL-6 concentration. The detection range of IL-6 was 4.69 pg/mL to 300 pg/mL, with a sensitivity of less than 0.3 pg/mL.

### HSA quantification and analysis by chromatography

Oxidized (HNA) and reduced HMA forms of HSA were analyzed using high-performance liquid chromatography (HPLC). HMA contains a reactive sulfhydryl group at position 34 (Cys34), while HNA is a mixed disulfide with glutathione or cysteine, sulfinic acid, sulfenic acid, or sulfonic acid. HPLC analysis was performed using a Shodex Asohipak ES-502 N column with a linear gradient elution of ethanol in a solution containing 0.05 mol/L sodium acetate and 0.4 mol/L sodium sulfate at pH 4.85. The oxidized albumin ratio (HNA/HMA) was determined as a marker of oxidative damage [14,15].

### Analysis of superoxide dismutase (SOD)

Superoxide dismutase (SOD) levels were measured using a human Cu/Zn SOD ELISA kit. The assay involved capturing human Cu/Zn SOD in serum with an anti-human Cu/Zn SOD antibody-coated microwell. An HRP-conjugated anti-human Cu/Zn SOD antibody was then added, followed by a substrate. The reaction was terminated with acid, and the absorbance was measured at 450 nm. The formation of a colored product was directly proportional to the concentration of human Cu/Zn SOD in the sample.

### Statistical analysis

Statistical analysis was performed using SPSS 23.0. Quantitative data were expressed as mean ± SD. Comparisons between means were made using the Wilcoxon signed rank test, with significance at *P* <0.05. Pearson's correlation coefficient was used to assess the relationship (r) between variables, and logistic regression analysis was used to predict the outcome. A correlation coefficient (r) measures the strength and direction of the relationship between two variables, ranging from -1 to +1, with +1 indicating a perfect positive correlation, 0 indicating no correlation, and -1 indicating a perfect negative correlation.

## RESULTS

**Figure 2:**
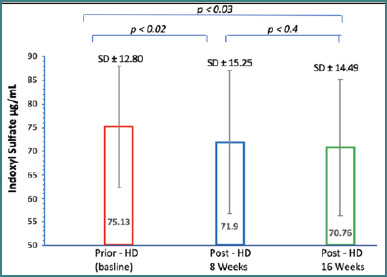
Quantification of serum IS levels using sandwich ELISA-based methodology. Serum IS levels were measured in 150 clinical samples taken prior to hemodialysis and at 8- and 16-weeks post-HD using commercial ELISA kits. The data were presented as means ± SD, and the significance of differences between the IS levels was evaluated using the Wilcoxon signed-rank test.

A total of 50 hemodialysis patients (36 men and 14 women) were included in the study, with ages ranging from 26 to 62 years (mean age 48.67 ± 8.15). The data consistently showed a significant reduction in IS levels during the treatment period. After 8 weeks, the mean level decreased to 71.9 ± 15.25 µg/ml, and after 16 weeks, it further decreased to 70.76 ± 14.49 µg/ml. Statistical analysis confirmed the significance of these reductions at both 8 weeks (*P* <0.023) and 16 weeks (*P* <0.034). However, there was no statistically significant difference between the 8-week and 16-week measurements (*P* = 0.494) (Figure 2). In addition, there was a significant and consistent reduction in creatinine and urea levels throughout the treatment period. At 8 weeks (creatinine: 5.93 ± 1.65 mg/dl, *P* = 0.0001; urea: 131.98 ± 39.53 mg/dl, *P* = 0.0001) and 16 weeks (creatinine: 4.36 ± 1.51 mg/dl, *P* = 0.0001; urea: 63.93 ± 19.35 mg/dl, *P* = 0.0001) of dialysis therapy, the levels were significantly lower compared to baseline measurements (creatinine: 9.03 ± 1.94 mg/dl; urea: 253.90 ± 68.01 mg/dl).

### Determination of inflammatory markers

The levels of CRP and IL-6 showed significant reductions at both 8 weeks (CRP: 21.39 ± 7.42 mg/l, *P* = 0.13; IL-6: 25.66 ± 9.83 pg/ml, *P* = 0.0001) and 16 weeks (CRP: 14.39 ± 5.91 mg/l, *P* = 0.0001; IL-6: 18.27 ± 7.73 pg/ml, *P* = 0.0001) of dialysis treatment compared to pretreatment levels (CRP: 23.53 ± 7.12 mg/l; IL-6: 34.27 ± 6.58 pg/ml). The serum levels of CRP and IL-6 were reduced by 11.7% and 7.68% at 8 weeks and 30.5% and 18.14% at 16 weeks of treatment, respectively (Table 1).

**Table 1:** Changes in biochemical and inflammatory markers pre and post-HD in patients with CKD

Serum Marker (Unit)	Prior-HD Mean ± SD	Post-HD		*P* value
		8 weeks Mean ± SD	16 weeks Mean ± SD	
**Creatinine (mg/dl)**	9.03 ± 1.94	5.93 ± 1.65^*^	4.36 ± 1.51^†‡^	0.0001^*^ 0.0001^†^ 0.01^‡^
**Urea (mg/dl)**	253.90 ± 68.01	131.98 ± 39.53^*^	63.93 ± 19.35^†‡^	0.0001^*†‡^
**CRP (mg/l)**	23.53 ± 7.12	21.39 ± 7.42^*^	14.39 ± 5.91^†‡^	0.138^*^ 0.0001^†‡^
**IL-6 (pg/ml)**	34.27 ± 6.58	25.66 ± 9.83^*^	18.27 ± 7.73^†‡^	0.0001^*†‡^

Data expressed as mean ± SD

Markers: CRP, C Reactive Protein; IL-6: Interleukin 6

The comparison of means was evaluated using the Wilcoxon Signed Rank test at three different time points: prior to HD compared to 8 weeks post HD*, prior to HD compared to 16 weeks post HD†, and post-HD 8 weeks compared to 16 weeks post HD‡. The serum level of CRP exhibited a reduction of 11.7% at 8 weeks and 30.5% at 16 weeks of treatment. Additionally, the serum level of IL-6 decreased by 7.68% at 8 weeks and 18.14% at 16 weeks of treatment.

### Assessment of oxidative stress biomarkers in serum

Table 2 presents the serum levels of oxidative stress biomarkers. After 8 weeks and 16 weeks of dialysis treatment, the serum levels of the antioxidant SOD significantly increased (*P* = 0.001) to 614.07 ± 146.27 ng/ml and 601.53 ± 98.87 ng/ml, respectively, compared to the pretreatment level (374.93 ± 92.96 ng/ml). The oxidative status of serum albumin was assessed using HPLC before and after dialysis therapy. The percentage of oxidized albumin (HNA%) showed a significant decrease (*P* = 0.0001) at both 8 weeks (47.67 ± 4.31) and 16 weeks (45.29 ± 2.23) posttreatment compared to the pretreatment level (62.356 ± 4.3). Conversely, the percentage of reduced albumin (HMA%) significantly increased (*P* = 0.001) at 8 weeks (51.29 ± 4.49) and 16 weeks (45.12 ± 3.89) after HD treatment compared to the pretreatment level (38.16 ± 2.42). However, the ratio of oxidative albumin (HNA/HMA) was significantly reduced (*P* = 0.0001) only at 16 weeks (0.96 ± 0.12) of dialysis treatment compared to the pretreatment level (1.71 ± 0.28). These findings suggest that dialysis treatment can improve oxidative stress in HD patients by increasing SOD levels and decreasing the HNA/HMA ratio.

**Table 2:** Serum oxidative stress markers and HSA status prior-HD and post-HD in patients with CKD

Serum marker (Unit)	Prior-HD Mean± SD	Post-HD		*P* value
		8 weeks Mean± SD	16 weeks Mean± SD	
**SOD (ng/mL)**	374.93 ± 92.96	614.07 ± 146.27^*^	601.53 ± 98.87^†‡^	0.0001^*†‡^
**HNA %**	61.58 ± 4.30	47.67 ± 4.3^*^	45.29 ± 2.23^†‡^	0.0001^*†^ 0.01^‡^
**HMA %**	38.16 ± 2.42	51.29 ± 4.49^*^	45.12 ± 3.89^†‡^	0.0001^*†‡^
**Albumin HNA/HMA ratio**	1.71 ± 0.28	0.98 ± 0.18^*^	0.96 ± 0.12^†‡^	0.0001^*†^ 0.314^‡^

Data expressed as mean ±SD

Markers: SOD, Superoxide dismutase; HNA, Human non-mercaptoalbumin; HMA, Human mercaptoalbumin; HNA/HMA, Oxidized albumin ratio. The comparison of means was evaluated using the Wilcoxon Signed Rank test at three different time points: prior to HD compared to 8 weeks post HD*, prior to HD compared to 16 weeks post HD†, and post-HD 8 weeks compared to 16 weeks post HD‡.

### Correlation between biochemical markers and indoxyl sulfate levels

**Figure 3:**
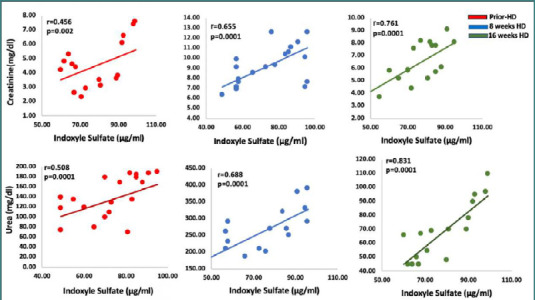
Correlation between biochemical markers with indoxyl sulfate levels before HD, after 8 weeks and 16 weeks post-HD. A correlation coefficient (r) measures the strength and direction of the relationship between two variables. It ranges from -1 to +1, where +1 indicates a perfect positive correlation, 0 indicates no correlation, and -1 indicates a perfect negative correlation.

The correlation between biochemical, inflammatory, and oxidative markers and indoxyl sulfate levels is shown in Figure 3. Throughout the treatment period, an association between indoxyl sulfate and creatinine and urea levels was consistently demonstrated in patients undergoing hemodialysis. Prior to treatment, the correlation coefficient for creatinine levels was 0.456 (*P* < 0.002), while the correlation coefficient for urea levels was 0.508 (*P* < 0.001), indicating a moderate association in both cases. The correlation coefficient between indoxyl sulfate and creatinine levels increased from 0.456 (*P* <0.002) to 0.655 (*P* < 0.001) following 8 weeks of treatment. Moreover, after 16 weeks of treatment, it increased to 0.761 (*P* < 0.001). Similarly, the correlation coefficient between indoxyl sulfate and urea levels increased from 0.508 to 0.688 after 8 weeks of treatment and increased further to 0.831 after 16 weeks. These findings indicate that as the treatment duration increased, the correlation between indoxyl sulfate and creatinine and urea levels strengthened (*P* < 0.0001).

### Correlation between inflammatory markers with indoxyl sulfate levels

Indoxyl sulfate levels were positively correlated with the levels of CRP and IL-6, meaning that higher indoxyl sulfate levels were associated with higher levels of inflammation. During the pretreatment period, the correlation coefficient for CRP levels was 0.575, while the correlation coefficient for IL-6 levels was 0.613, indicating a moderate association in both cases (*P* < 0.0001). This implies that patients with higher indoxyl sulfate levels tended to exhibit higher CRP and IL-6 levels.

**Figure 4:**
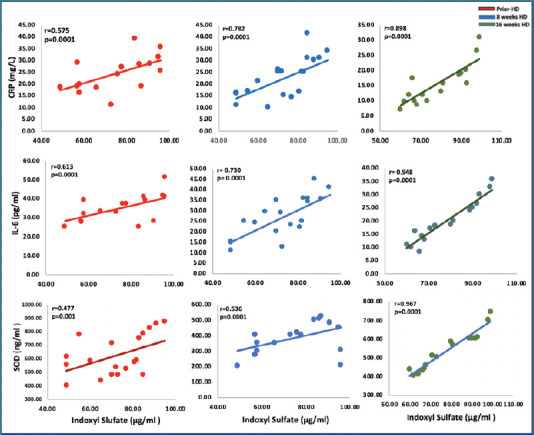
Correlation between inflammatory and antioxidant markers with indoxyl sulfate levels prior HD, after 8 weeks and 16 weeks post HD

The correlation coefficients of the relationship between indoxyl sulfate and CRP and IL-6 levels demonstrated a notable increasing trend. After 8 weeks of treatment, the correlation coefficients increased to 0.782 for CRP and 0.730 for IL-6. Following 16 weeks of treatment, these coefficients increased to 0.898 for CRP and 0.948 for IL-6. These findings strongly suggest a progressively stronger positive association between indoxyl sulfate and both CRP and IL-6 levels as the treatment advances (*P* < 0.0001), as shown in Figure 4.

### Correlation between antioxidant marker (SOD) and indoxyl sulfate levels

SOD is a crucial antioxidant enzyme that plays a vital role in neutralizing ROS and safeguarding cells against oxidative damage. The accumulation of IS could result in increased oxidative stress, potentially impacting the activity or expression of SOD. Consistently, the findings indicate an association between IS and SOD levels throughout the treatment period. The correlation coefficients progressively increased in magnitude over the treatment period. These values started at 0.477 before treatment, increased to 0.536 after 8 weeks, and increased to 0.967 after 16 weeks. These results suggest a continuous strengthening of the positive relationship between IS and SOD levels as the treatment progresses (*P* < 0.0001, Figure 4). These findings could be explained by the possibility that the reduction in indoxyl sulfate may be linked to an elevation in the levels of the antioxidant SOD and a decrease in inflammation.

### Correlation between oxidative markers and indoxyl sulfate levels

**Figure 5:**
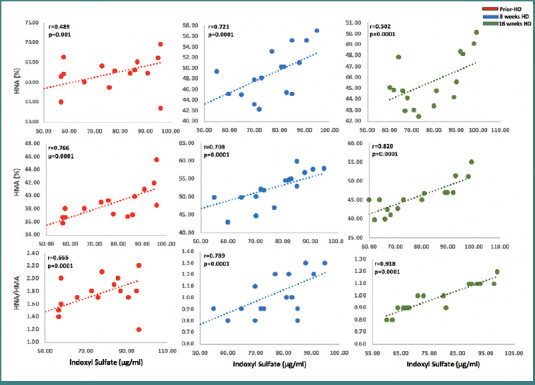
Correlation between IS levels, oxidized albumin, and HNA/HMA ratio prior HD, after 8 weeks and 16 weeks post HD

The oxidative albumin ratio, specifically the HNA/HMA ratio, measures oxidative stress in the body. It represents the ratio of high molecular weight albumin (HMA) to low molecular weight albumin (HNA), with higher values indicating increased oxidative stress. The elevated levels of indoxyl sulfate have been associated with increased oxidative stress, potentially influencing the HNA/HMA ratio. Indoxyl sulfate might alter the balance between high-molecular-weight and low-molecular-weight albumin, leading to changes in the oxidative albumin ratio. The correlation coefficients provide valuable insights into the association between indoxyl sulfate and the oxidative albumin ratio and its components. Prior to treatment, a relationship was found between indoxyl sulfate levels and HNA levels (*r* = 0.489), HMA (*r* = 0.766), and the oxidative HNA/HMA ratio (*r* = 0.655), indicating a potential link between indoxyl sulfate and increased oxidative stress (*P* = 0.0001). During the treatment period, the correlation coefficients had a consistent strengthening of the positive relationship between IS and HNA, HMA, and the oxidative HNA/HMA ratio (*P* = 0.0001). This suggests that the influence of indoxyl sulfate on oxidative stress levels intensifies over time, as reflected in the changes observed in the albumin components and the oxidative albumin ratio (Figure 5).

## DISCUSSION

In the present study, we examined and compared the levels of IS in individuals with CKD undergoing hemodialysis treatment and those not receiving hemodialysis. Additionally, the study aimed to explore the relationships between IS and markers of renal function, inflammation, and oxidative stress.

IS is a uraemic toxin produced by gut bacteria during the breakdown of tryptophan to indole. More than 90% of IS in the circulation is associated with plasma proteins removed by tubular secretion in the native kidney [16]. However, because only free unbound IS diffuses across the dialysis membrane, dialysis clearance of blood IS levels in patients with CKD is limited [17]. The accumulation of IS in patients with CKD is associated with worsening renal function, increased inflammation, and heightened oxidative stress, all of which contribute to the progression of end-stage renal disease (ESRD) and CVD [16]. Moreover, elevated IS levels have been linked to an increased risk of cardiovascular mortality in patients with CKD. [18,19]. As a result, serum IS levels may be helpful in identifying problems in individuals with end-stage kidney disease. The routine measurement of IS levels may aid in determining treatment efficacy, such as dialysis or a combination strategy to lower IS levels [20], and predict disease progression in patients with CKD.

In the bloodstream, over 90% of IS binds to plasma proteins, and under normal kidney function, these protein-bound IS molecules are eliminated through tubular secretion in the kidneys [21]. However, in individuals with CKD, dialysis struggles to efficiently clear IS from the serum because only the unbound, free form of IS can diffuse across the dialysis membrane [17]. This predicament results in elevated IS levels among hemodialysis patients. The buildup of IS in patients with CKD aggravates kidney function, inflammation, and oxidative stress, potentially propelling the advancement of end-stage renal disease and cardiovascular issues [16]. Notably, increased IS levels are linked to both overall mortality and cardiovascular-related deaths in those with CKD [18].

Our study has provided evidence to support the hypothesis that the serum concentration of indoxyl sulfate is significantly increased in the prior hemodialysis group. However, the study revealed a substantial decrease in serum indoxyl sulfate levels throughout the treatment duration (*P* < 0.023 and *P* < 0.034 at 8 and 16 weeks, respectively). Furthermore, the data showed a notable and continuous decline in renal function levels throughout the treatment duration among individuals undergoing hemodialysis. According to the findings of Lin *et al*., there was a gradual increase in serum indoxyl sulfate levels among dialysis patients as their renal function declined during hemodialysis [22]. Our study revealed a significant correlation between blood indoxyl sulfate levels and renal function levels among patients undergoing hemodialysis treatment (*P* < 0.0001). This is consistent with previous studies, which have shown that IS levels are positively correlated with urea and creatinine levels in patients with CKD undergoing dialysis. The correlation exists because IS, like urea and creatinine, is normally cleared by the kidneys; however, dialysis is less efficient at removing IS compared to urea and creatinine, leading to persistently higher IS levels in patients undergoing dialysis [6,23]. According to a wide search of other previous studies, our study is significant because it is the only study to describe the correlation between IS, urea, and creatinine and specific time intervals in patients with CKD. This provides new information about how these biomarkers change over time in patients with CKD.

Consequently, the level of IS in the bloodstream could serve as an indicator of complications in individuals with advanced kidney disease. Ultimately, regularly assessing IS levels may prove valuable in gauging the efficacy of treatments such as dialysis or a combined approach to reduce IS levels [20]. Moreover, such evaluations could offer insights into predicting disease progression in patients with CKD.

It is well known that oxidative stress and inflammation are both common in people with CKD. These processes are thought to be interconnected, possibly causing the other. Both conditions can lead to endothelial dysfunction, a problem with the lining of blood vessels that can lead to CVD and other complications. In patients undergoing stable maintenance hemodialysis, inflammatory status and the duration of HD have been reported as significant predictors of overall survival [24]. In CKD, the precise relationship between oxidative stress and inflammation remains unclear. However, it is believed that they are connected in various ways. Oxidative stress can induce inflammation through several mechanisms. First, ROS can directly activate inflammatory signaling pathways. Second, ROS can damage cells, leading to the release of pro-inflammatory cytokines. Third, ROS can modify biomolecules, such as lipids and proteins, making them more susceptible to attack by immune cells. In order to enhance the patient's overall health, it is necessary to manage these conditions [25].

In the current study, CRP and IL-6 levels decreased significantly after 8 weeks (*P* = 0.13 and *P* = 0.0001, respectively) and 16 weeks (*P* = 0.0001 and *P* = 0.0001, respectively) of dialysis treatment, when compared to the levels before dialysis. Numerous studies have found an association between renal impairment and various inflammatory mediators and markers, specifically CRP and IL-6, even in patients with moderate renal impairment, indicating that CKD is a low-grade inflammatory process [26]. Nasif *et al*. hypothesized that CRP and IL-6 serum levels were reduced by 11.7% and 7.68% after 8 weeks of treatment and by 30.5% and 18.14% after 16 weeks [27].

SODs play a critical role in the body’s antioxidant defense system against oxidative stress. Our results confirmed that there was a significant correlation between the level of SOD after 8 weeks and 16 weeks of dialysis treatment compared to those prior treatment (*P* = 0.001). Numerous studies have demonstrated the therapeutic potential and physiological importance of SOD. This enzyme can act as an anti-inflammatory agent and prevent precancerous cell transformations [28]. The body’s natural SOD levels decline with age [29], and as a result, aging increases susceptibility to oxidative stress-related diseases. Oxidative stress is a state in which there is an imbalance between the production of reactive oxygen species and the body's ability to neutralize them. This imbalance can damage cells and tissues and is a major contributing factor to chronic kidney disease. Oxidized albumin is a form of albumin that has been damaged by ROS. It is a marker of oxidative stress and has been shown to be associated with a number of adverse outcomes in CKD, including progression of kidney disease, cardiovascular disease, and mortality [30].

Human serum albumin (HSA) has 585 amino acids. It weighs 66 kDa and is produced by the liver. HSA has 35 cysteine residues, although only cysteine 34 is unbonded. This free cysteine residue in HSA scavenges free radicals. HSA occurs in two forms: oxidized HNA and reduced HMA [31,32]. HNA and HMA can reflect systemic oxidative stress in chronic liver failure and patients with CKD [33,34]. The HNA/HMA ratio is a marker of oxidative stress, an imbalance between the production of free radicals and the body's ability to neutralize them. High HNA levels are a substantial risk factor for cardiovascular disease in patients undergoing hemodialysis and peritoneal dialysis. Evaluation of HNA and HMA in patients with CKD at high risk of CVD is clinically useful.

The current study assessed the oxidative status of serum albumin using HPLC before and after dialysis therapy. A significant decrease at both 8 weeks and 16 weeks post-dialysis compared to pre-dialysis was observed (*P* = 0.0001). This is concomitant with significantly reduced albumin levels (HMA%) (*P* = 0.001) at 8 weeks and 16 weeks after HD treatment compared to the pre-dialysis level. These findings emphasize that increased SOD levels and a decreased HNA/HMA ratio are also indicators of reduced oxidative stress in HD patients, as seen in this study (*P* = 0.0001). According to the findings of Nasif *et al*., both HNA and HMA levels were significantly lower (*P* = 0.001) after 8 weeks of treatment, but the oxidized albumin ratio HNA/HMA was significantly (*P* = 0.001) lowered only at 16 weeks [27]. Other studies demonstrated that patients with higher levels of oxidized albumin had a greater proportion of mortality attributable to cardiovascular complications, as well as a higher overall mortality rate [34,35].

Studies have shown that IS can induce oxidative stress and increase the production of HNA and HMA. IS can also bind to HSA and inhibit its antioxidant activity. This can lead to further oxidative stress and damage to cells and tissues [27]. Studies have shown that IS levels are correlated with HNA levels in patients with CKD. This suggests that IS may contribute to oxidative stress in patients with CKD by increasing HNA levels. Our results confirmed an association between indoxyl sulfate and HNA, HMA, and the oxidative HNA/HMA ratio (*P* = 0.0001). Additionally, IS levels were significantly correlated with f (HNA), a marker of HNA levels. Nakatani *et al*. also found that IS levels were associated with a number of other adverse outcomes in patients with CKD, including anemia and cardiovascular disease [36]. Additionally, IS, HNA, and HMA have all been related to a higher risk of cardiovascular disease in patients with CKD due to their high contribution to the development of atherosclerosis [36,37].

This study has a number of limitations. First, it is a prospective, observational cohort study, meaning we could only evaluate the relationships between variables at one point. This makes establishing causal relationships between the variables and selection bias impossible. Second, the study has a relatively small sample size, which limits the power of the results. Third, the study participants were all patients with CKD, so the results may not be generalizable to other populations. Despite these limitations, this study provides valuable information about the relationships among IS, kidney function, inflammation, and oxidative stress in patients with CKD. Further research is needed to confirm these findings and to investigate the causal mechanisms underlying the observed associations.

## CONCLUSION

In conclusion, this study investigated the potential associations between IS, HNA, and HMA in the development and progression of CKD, as well as its complications. Reducing IS and HNA/HMA levels may be a therapeutic target for improving outcomes in patients with CKD. Additionally, our findings revealed associations between IS and markers of renal function, inflammation, and oxidative stress in patients with CKD. These findings indicate an association between IS and the progression of CKD and its associated complications. The current results are highly supported. Further research is needed to investigate the exact mechanisms by which IS contributes to these complications and to develop effective therapies to reduce IS levels in patients with CKD undergoing dialysis.

## Data Availability

The data that support the findings of this study are available from the corresponding author upon reasonable request.
